# Nonalcoholic Steatohepatitis: A 9-Year Follow Up Cohort Study

**DOI:** 10.3390/jcm11195969

**Published:** 2022-10-10

**Authors:** Alessandra Mangia, Annarita Valeria Piazzolla, Maria Maddalena Squillante, Giovanna Cocomazzi, Vanna Maria Valori, Massimiliano Copetti, Paola Parente, Vito Attino, Maria Guido

**Affiliations:** 1 Liver Unit, IRCCS Fondazione “Casa Sollievo della Sofferenza”, 71013 San Giovanni Rotondo, Italy; 2 Oncology Division, IRCCS Fondazione “Casa Sollievo della Sofferenza”, 71013 San Giovanni Rotondo, Italy; 3 Ostatistics Department, IRCCS Fondazione “Casa Sollievo della Sofferenza”, 71013 San Giovanni Rotondo, Italy; 4 Pathology Division, IRCCS Fondazione “Casa Sollievo della Sofferenza”, 71013 San Giovanni Rotondo, Italy; 5 Department of Pathology, Azienda ULSS2, 31100 Treviso, Italy; 6 Department of Medicine, University of Padua, 35131 Padua, Italy

**Keywords:** non-invasive diagnosis, cirrhosis, fibrosis, NASH, liver histology

## Abstract

Background and aim: Non-alcoholic fatty liver disease (NAFLD) may progress to severe liver fibrosis and cirrhosis. A limited number of studies with a long follow up assessed fibrosis progression and related predictors in untreated patients with a histological diagnosis of NAFLD. This study aims to investigate rate and predictors of NAFLD progression. Methods: For 9 (2–16.7) years, we followed up a cohort of patients histologically diagnosed. Disease progression was defined by a composite endpoint as evidence of cirrhosis in patients without cirrhosis at baseline, evidence of de novo occurrence of cirrhosis complications, histologically established worsening of stage 1 of fibrosis or increase of 20% in liver stiffness by transient elastography in patients rejecting a second liver biopsy. Results: A total of 91 patients were enrolled. Of them, 31 had NAFL and 60 NASH. A second liver biopsy was performed in 22 NASH patients and in 4 NAFL. Disease progression was observed in 38.5% NASH and in 12.0% NAFL (*p* = 0.034). Patients with portal inflammation had a higher risk of progression (66.7% vs 26%, *p* = 0.021). High triglycerides levels, advanced fibrosis at baseline and the duration of follow-up predict disease progression (*p* = 0.021; OR = 6.93, 95% CI 1.33–36.08, *p* = 0.43; OR 8.37; 95% CI 1.07–65.58 and *p* = 0.034; OR = 0.88; 95% CI 0.78–0.99, respectively). Conclusions: Our results reinforce the evidence that, in the absence of pharmacologic treatment, NASH progresses in about 40% of patients. Liver biopsy is the only mean to discriminate NAFL from NASH. The prognostic role of portal inflammation needs to be explored in larger series.

## 1. Introduction

Non-alcoholic fatty liver disease (NAFLD) encompasses conditions such as non-alcholic fatty liver (NAFL), non-alcoholic steatohepatitis (NASH), fibrosis, and cirrhosis, that may require a histopathologic definition. Both a 25% prevalence in the general population, and the rates of NASH-related HCC and liver transplantation, are expected to double by 2030, explaining the growing interest in this condition [1,2,3].

Liver biopsy allows distinction between NAFL and NASH, and the stages of fibrosis severity and disease activity [2,4]. However, it is expensive and invasive, with small but significant risk of complications including a 0.35% risk of bleeding and a 0.14% risk of death [5]. Moreover, accurate histological diagnosis requires expert pathologists and, despite this, intra- and interobserver agreement represents a critical aspect of diagnosis [6]. Finally, subtle differences between the different histological scoring systems exist [7,8]. Nevertheless, EASL guidelines on non-alcoholic fatty liver and, more recently, guidelines on non-invasive tests for the evaluation of NAFLD severity and prognosis, state that the identification of advanced fibrosis (F3) or cirrhosis (F4) by non-invasive serum biomarkers (NITs) and transient elastography (TE) in NAFLD, is less accurate than using liver biopsy [9,10].

Several studies investigated the natural history of liver disease in NAFLD; however, the longitudinal assessment of fibrosis progression on multiple biopsies is hardly accepted in the real world and will become even more difficult in the near future, with the approval of experimental drugs under evaluation in phase II and III clinical trials. In a series of 70 patients with paired biopsies, Pais et al. showed that a substantial proportion of subjects progress from NAFL to NASH after 3.4 years [11]. In a meta-analysis of 11 studies with paired biopsies, Singh et al. demonstrated in 411 patients with NAFLD, stage 1 of fibrosis progression after 7.1 years, among patients with NASH [12]. Moreover, disease specific mortality risk was evaluated by Ekstedt et al., in a longitudinal study on 229 biopsy-proven NAFLD, after a mean follow up duration of 26.4 years. Patients with fibrosis stage 3 or 4 at baseline had increased mortality (HR 3.3, *p* < 0.001), irrespective of NAS score [13]. More recently, all NAFLD histological stages have been associated with significantly increased overall mortality [14].

As fibrosis does not increase linearly, diagnostic, and prognostic relevance of stage 2 diagnosis has been highlighted [15], making liver histology indispensable in clinical trials on developing therapeutical compounds. Indeed, data on the non-invasive assessment of fibrosis in NASH [16] show non-invasive biomarkers to be sensitive in excluding fibrosis stage ≥ 3, but not sufficiently specific to diagnose cirrhosis, nor able to accurately discriminate between different fibrosis stages or between NAFLD and NASH [10]. Among non-invasive testing, the Fibrosis-4 index (FIB-4) is cheap, feasible, and associated with accuracy for the diagnosis of significant fibrosis (AUROC 0.79) and cirrhosis (AUROC 0.80) [17]. As an imaging non-invasive method, transient elastography (TE) is rapid and has a good negative predictive value (NPV), but may be limited in obese patients if the XL probe is not available. Moreover, the prognostic significance of over time changes in liver stiffness has been evaluated in several studies but, only in a few of them, in the context of NAFLD [18].

We longitudinally followed up a cohort of Italian patients with histological diagnosis of NASH, with the aim of assessing the proportion of untreated patients who progress over time, and of identifying the predictors of disease progression.

## 2. Methods

### 2.1. Baseline Evaluation

**Figure 1:**
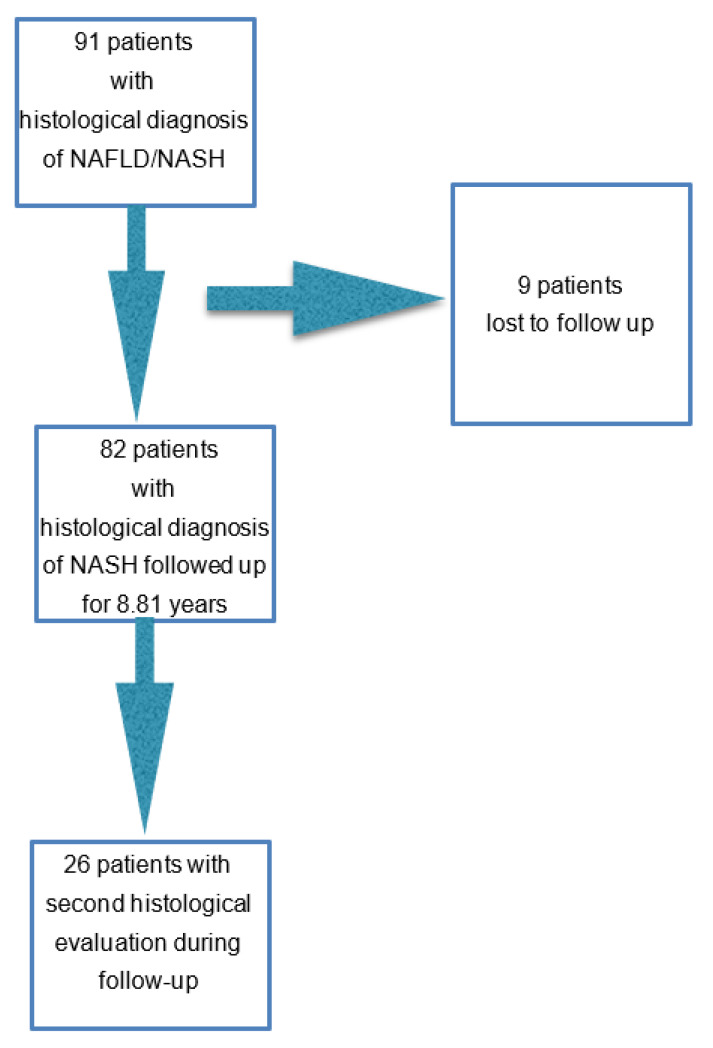
Study flow chart showing the number of patients initially included, the number of subjects lost to follow up and the number of patients with paired biopsies. (NAFLD = Non-Alcoholic fatty liver disease; NASH = Non-alcoholic steatohepatitis).

Ninety-one patients with a consecutive histological diagnoses of NAFLD, obtained from a larger pool of patients with clinical suspicion from January 2001 to December 2008, and as part of an investigation on abnormal liver enzymes, were included in this longitudinal study (Figure 1). They were patients with NAFLD in the absence of secondary causes. The criteria for the presumptive diagnosis of NAFLD were: increased levels of ALT, AST, GGT, and radiological evidence of steatosis daily alcohol intake of less than 40 g for men and less than 20 g for women.

A diagnosis of diabetes was established based on stable antidiabetic treatment or fasting plasma glucose test results > 126 mg/dL [19]. Hyperlipidemia was diagnosed on fasting cholesterol levels > than 200 mg/dL or triglyceride levels > than 150 mg/dL. Obesity was defined by kg of body weight/m^2^ body mass index (BMI) > 30 both in men and women. A history of systemic hypertension (HBP), defined in accordance with the American College of Cardiology [20], was available in all, as well as waist circumference. After diagnosis, patients were offered follow-ups every 6 months. Patients lost to follow-up were contacted by phone calls or conventional mail to keep them adherent to the initial monitoring plan.

Liver biopsy samples were obtained using the modified Menghini technique (Biomol 16 or 18 G). One or two passes were performed. Formalin-fixed, paraffin embedded liver sections were stained routinely with hematoxylin and eosin, and Masson trichrome for collagen. All liver specimens were considered adequate, with a minimum number of portal spaces = 10. They were re-examined in a blind and non-paired manner by an experienced liver pathologist (MG), who was unaware of clinical and biochemical data of patients. An histological diagnosis of NASH was based on accepted criteria [2,4] requiring the presence of any degree of hepatocyte ballooning and lobular inflammation, in addition to macrovesicular steatosis. Biopsies were graded according to the SAF scoring system (steatosis, activity, fibrosis) [7]. Steatosis (S) was scored from 0 to 3 (S0: <5% of the liver parenchyma; S1: 5–33%, mild; S2: 34–66%, moderate: S3: >67%, marked). Activity grade (A) was obtained by summing hepatocyte ballooning (0–2) and lobular inflammation (0–2) and defined as mild (A1), moderate (A2), and severe (A3). Stage of fibrosis (F) was assessed using the NASH-CRN score [7]. In addition, the degree of portal inflammation, not included in the SAF score, were separately evaluated on a 0 to 3 scale (absent to severe) [7]. NAFL was defined as steatosis only [2,4,7].

During the follow up, portal hypertension endoscopic evaluation was performed per BAVENO VI criteria [21].

The variation of PNPLA3 (patatin-like phospholipase domain containing 3, gene) rs738409 on long arm of chromosome 22 was assessed in every patient as described [22].

#### 2.1.1. Follow Up

Patients were followed up with a biannual clinical and biochemical evaluation and underwent TE evaluation and/or liver biopsy during follow up when they failed lifestyle intervention. The mean follow up between baseline biopsy, and last follow up assessment, was 8.81 ± 0.8 years (median 5, range 1 to 21 years). Only 26 agreed to a second biopsy. Patients on follow up were also evaluated by TE bi-monthly from 2007 (year of Fibroscan availability) to 2021.

During follow up, 6 patients with a diagnosis of fibrosis 3 or 4 at baseline developed esophageal varices and 5 died due to stroke or non-liver cancer, with a single case of HCC.

#### 2.1.2. Study Endpoint

The primary endpoint of this longitudinal analysis was disease progression. Disease progression was defined, by a composite endpoint, as evidence of cirrhosis in patients without cirrhosis at baseline, evidence of the de novo occurrence of cirrhosis complications, histologically established worsening of stage 1 of fibrosis or an increase of 20% in liver stiffness by TE in patients rejecting a second liver biopsy.

### 2.2. Statistical Analysis

Baseline demographical and clinical characteristics were reported as mean ± standard deviation or median and range for continuous variables, and as frequency and percentages for categorical variables.

Group comparisons were carried out using the exact Wilcoxon rank-sum test for continuous variables and Pearson chi-square or Fisher test, as appropriate, for categorical variables.

In the basal liver biopsy, in addition to the fibrosis stage and grade of activity, the severity of steatosis and grade, the type (portal/lobular) of inflammation and presence or absence of ballooning, were included among the variables examined.

As potential predictors of fibrosis, in addition to the histological ones, the following variables were considered: age, gender, diabetes, total and LDL cholesterol, triglycerides, obesity, PLT count, AST, ALT, GGT, and Fib-4 index.

Patients were grouped into “progressors” and “non-progressors”. Disease progression was defined as: (i) worsening of at least 1 point on histological fibrosis score; (ii) worsening of liver stiffness results (13); and (iii) occurrence of clinically evident liver cirrhosis or liver related complications as esophageal varices in patients without varices at baseline.

An analyses on the potential predictors of progression were performed: (i) on all 91 patients; and (ii) in the sub-group of 26 patients who agreed to a second liver biopsy.

A *p*-value < 0.05 was considered statistically significant. All analyses were performed using the SPSS v.15 (SPSS, Chicago, IL, USA) and the SAS Release 9.4 (SAS Institute, Cary, NC, USA).

## 3. Results

The baseline characteristics of 91 patients followed up for 8.8 years are reported in Table 1. At baseline, 31 (34.1%) had NAFL and the remaining 60 (65.9%) NASH. The population included 47.2% male, median age was 52.5 (range 20–79). Forty-five (49.4%) patients had diabetes, 48 (52.7%) were obese, 36 (40.0%) had cholesterol levels higher than 200 mg/dL, 33 (36.7%) had triglycerides levels higher than 150 mg/dL, and 19 (21.1%) had both. Thirteen (14.6%) had HBP. The median AST and ALT levels were 39.5 U/L (12–173) and 62.0 U/L (11–223), respectively. Median GGT level was 43.0 U/L (5–862).

In the basal liver biopsy, the grade of steatosis was mild in 36.4% of patients, moderate in 31.9%, and severe in 28.6%. According to the SAF score, the grade of activity was 0 in 14.3%, 1 in 47.3% of cases, 2 in 27.2%, and 3 in 11.0%. A portal inflammation of grade 0–1 was detected in 69.3%, and of grade 2 in 19.4% of patients.; a portal inflammation of grade 3, in 11.3%.

Basal fibrosis evaluation is reported in Table 1. At baseline, 24.4% of patients were in fibrosis stage 2, 30.5% in advanced fibrosis, and 14.6 in cirrhosis. The GG genotype codifying for methionine substitution in the PNPLA3 gene at position 148 was detected in 26.1% of patients.

### 3.1. Progression of Disease and Relative Predictors

Of 91, 9 (9.8%) patients -the majority of whom from the NAFL group—were lost to follow up or moved to another geographic area. Of the remaining 82, including 25 NAFL and 57 NASH, 22 (38.5%) with NASH showed disease progression as compared to three (12.0%) with NAFL. Of the five deaths from the NASH group, only one was liver related. In total, four patients developed endoscopic evidence of portal hypertension and esophageal varices, the remaining 16 had histological evidence of progression, five had evidence of 20% increase in liver stiffness. One of five NAFL patients who agreed to a second liver biopsy showed progression.

As shown in Table 1, no significant differences between “progressors” and “non-progressors” were found in gender, age, BMI, ALT, AST, or platelet count. Only diabetes, high triglycerides, and liver stiffness, were associated with progression. Although none of the patients was specifically receiving pharmacological therapies, all of them were encouraged to decrease their caloric and fat intake and increase exercise. During the follow-up, only four patients achieved a 5–7% reduction in their body weight, three of them were in the group of “non progressor” and one in the group of “progressors” (*p* = 0.63).

As for histological changes, only fibrosis stage >2, a portal inflammation of grade 3 (*p* = 0.018), and activity higher than 1 (*p* = 0.05), were associated with disease progression by univariate analysis. A numerically higher number of patients with severe lobular inflammation showed progression (*p* = 0.083). A FIB-4 higher than 2.67 at baseline was predictive of disease progression (*p* = 0.044).

The results of the multivariable regression analysis, also including the duration of follow up as an independent variable and adjusting for key demographic factors such as age, gender, and BMI, showed that high triglycerides levels, advanced fibrosis at baseline, and longer duration of follow-up, predict disease progression (*p* = 0.021; OR = 6.93, 95% CI 1.33–36.08, *p* = 0.43; OR 8.37; 95% CI 1.07–65.58 and *p* = 0.034; OR = 0.88; 95% CI 0.78–0.99, respectively).

### 3.2. Progression of Fibrosis

**Figure 2:**
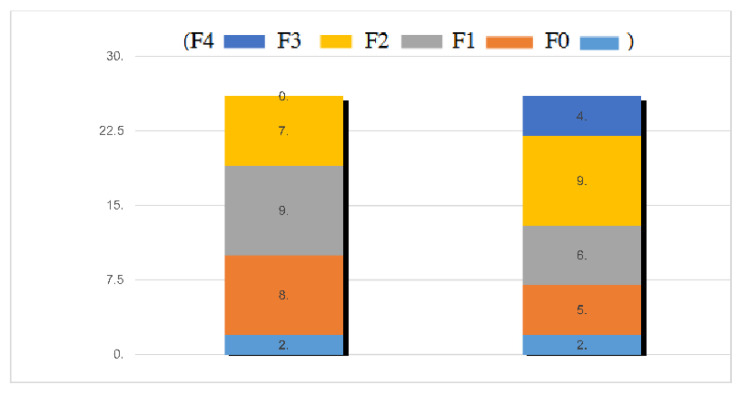
Fibrosis changes in 26 patients undergone a second liver biopsy. The first column indicates the number of patients with different fibrosis stages at baseline, the second the correstonding numbers at the follow up liver biopsy.

The second liver biopsy was performed after a median time of 8.31 years ± 1.41 (median 4, range 2–21 years) in the 26 patients who agreed to it. Histological features in the two paired biopsies are reported in Figure 2 and Table 2. At baseline, fibrosis stage 1 was present in 8 (30.7%), stage 2 in 9 (34.6%), stage 3 in 7 (26.9%); any patient had stage 4, and two patients had no fibrosis.

**Figure 3:**
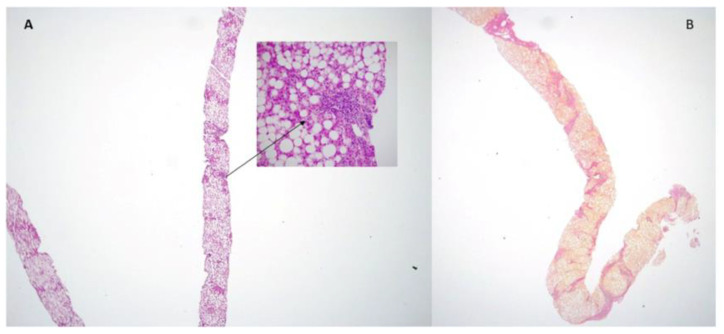
(**A**) NASH with severe steatosis and moderate portal inflammation (arrow) at presentation (H&E; original magnification 1.2×; insert original magnification 40×. (**B**) The same patient showed cirrhosis in the follow up biopsy performed 4 years later (Van Gieson’s stain; original magnification 2.5×).

Severe steatosis was present in seven patients (26.9%). Overall, steatosis tended to very marginally increase in severity over the follow up interval. Indeed, in only two out of seven patients with mild steatosis at baseline, a moderate steatosis was observed after the follow up. Activity was mild in 73.0% of cases, moderate in 19.3%, and severe in 7.7% of patients. At the end of follow up, the number of patients with moderate and severe activity substantially increased, as three patients progressed from mild to moderate and two from moderate to severe (Table 2). The number of subjects with grade 2 portal inflammation increased from 11.5% to 19.2%, and grade 3 from 0 to 3.8%. Figure 3 depicts individual changes in fibrosis among “progressors”. Notably, four out of five NAFL patients who received a second liver biopsy had fibrosis improvement, as compared to only two of 21 NASH patients (9.5%).

**Figure 4:**
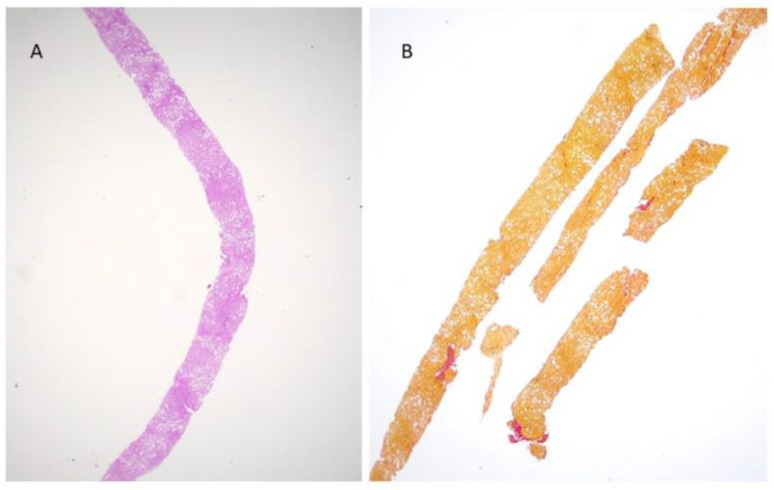
(**A**) Moderate steatosis with minimal lobular inflammation at presentation (H&E; original magnification 2.5×); (**B**) same patient 15 years later did not show any fibrosis progression (Van Gieson’s stain; original magnification 2.5×).

We failed to observe any correlation between fibrosis progression and clinical data, with the exception of triglycerides higher than 150 mg/dL. Indeed nine of 11 “progressors” had triglycerides higher than 150 mg/dL as compared to five out of 15 non-progressor (*p* = 0.021). As for diabetes, zero out of 13 patients without diabetes, and nine out of 13 with diabetes, experienced histological progression (*p* = 0.002). Among histological variables, both fibrosis and portal inflammation were associated with the progression of disease (*p* = 0.001 and *p* = 0.039, respectively). A significantly higher proportion of patients with advanced fibrosis (88.9%) experienced progression, as compared to only three of 17 subjects (17.6%) with stage 0–2 (*p* = 0.001). Finally, 27.2% of “progressors” had portal inflammation of grade 2–3 as compared to zero of “non-progressors” (*p* = 0.039). A multivariate analysis was not performed due to the small sample size. Figure 3 and Figure 4 depict histological changes in two of our patients with paired liver biospies after 4 and 11 years, respectively.

Among patients who had fibrosis progression, 16 (76.1%) carried PNPLA3 GG, but the rate was not statistically higher when compared to the 93.3% of non-progressors (*p* = 0.27).

## 4. Discussion

The results of this large monocentric series of histologically diagnosed NAFLD patients provide evidence that, in Southern Italy, about 40% of patients with NASH diagnosis progress to more severe liver disease, as compared to only 12% of those with simple steatosis, in a median follow-up period of about 9 years. Both rates confirm the progressive nature of this condition [12,23,24,25], while reinforcing the idea that unambiguous NAFL diagnosis at baseline is the hallmark of a benign prognosis. Differences in diabetes numbers across the cohorts, and a race effect, may account for slight variability across different cohorts. Indeed, Singh et al., in their meta-analysis, observed 34.5% worsening, 38.8% stabilizing, and 26.7% improving [12]. McPherson observed 42% progression, 40% no changes, and 18% improvement, in fibrosis [25]. Similar fractions of fibrosis progression (40.7%) were reported by Younussi in a global analysis of 729 NAFLD studies [23]. In histological paired biopsy studies [11,25], a low rate of progression has been reported for bland steatosis. The difference in progression rate between NAFL and NASH patients after a long-term follow-up in our study, confirm the possibility of relaxed liver monitoring in patients with NAFL, after an accurate baseline designation. Indeed, to either avoid unnecessary stressing procedures for patients who do not need close follow-up and monitoring, or to reduce the enormous burden of metabolic related liver diseases for physicians, it would be crucial to understand in advance what patients require close monitoring. Moreover, early diagnosis and lifestyle counseling can reduce progression to severe disease, or liver transplant, and lead to survival advantages for patients at risk of severe NASH [26].

In addition to a stage of fibrosis 3 or higher, in our series, activity grade and portal inflammation at baseline were both associated with disease worsening. These results suggest that disease evolution is influenced by histological activity. This observation was also confirmed in the setting of repeated liver biopsy; however, probably due to the limited sample size, activity did not result in an independent predictor of disease progression. This finding has great practical relevance. In fact, despite increased portal inflammation being known to be associated with clinical and pathologic features of progressive NAFLD in 728 adults and 205 children [27], it is neither a diagnostic criterion for NASH nor a component of any scoring system. Moreover, in the available studies demonstrating that portal inflammation is related to fibrosis, a cross sectional rather than a longitudinal evaluation of liver histology has been usually performed. Only recently, changes in activity have been shown able to influence fibrosis progression on the basis of 446 paired biopsies [28]. In keeping with this important and very large study, our results suggest that greater relevance should be given to portal inflammation into the specific definition of the grade of activity.

We suggest that, in selected patients with metabolic syndrome and risk factors for progressive liver disease, a baseline liver biopsy interpreted by a trained liver pathologist, and informative on severity of portal inflammation, provides relevant prognostic information.

Interestingly, the progression of fibrosis is a function of the duration of follow up, the longer the follow up duration the higher the risk of progression. In patients with NASH, progression seems to be inevitable. No demographic or biochemical tests, with the only exception of triglycerides, neither non-invasive indexes or stiffness results, were independent predictors of liver disease progression, in our study population. This holds true both in the whole group of patients longitudinally followed and in the subgroup of those with paired biopsies. Diabetes has been identified as predictive of severe fibrosis at diagnosis [29] and recently, a family history of diabetes has been associated with an increased risk of NASH [30]; in our series- probably due to the limited sample size- it did not result in an independent predictor. A PNPLA3I148M polymorphism due to the C—>G substitution has been associated with the increased proportion of fibrosis, steatosis, and NASH [31,32]. A PNPLA3 substitution was observed in about similar proportion of “progressors” and “non-progressors”. This is not unexpected as to date any genetic variant has transitioned into diagnostic tools [33].

The other question is how, and how frequently, our patients should be followed in real life to monitor changes in disease severity. It could be advised that once the presence of fibrosis is identified at baseline by liver biopsy, TE can be used bi-monthly or yearly in patients at risk over time. Indeed, although TE is less accurate in measuring fibrosis in fatty liver than in patients with viral hepatitis [34], it has recently been demonstrated able to predict clinical outcomes in NAFLD [18]. On the other hand, we confirm the limited prognostic role of other serum markers, including FIB-4 index. Other recently validated algorithms, based on biomarkers as alpha2macroglobulin, HA, and TIMP1, are associated with better performances in discriminating between patients with advanced fibrosis or cirrhosis. These tests are costly and not available everywhere [35]. In our study, FIB-4 was used only as a tool to select liver biopsy candidates expected to have some degree of fibrosis, while TE with both M and XL probes was used since 2017 to monitor our patients during the follow-up, regardless of their choice to receive a second liver biopsy. TE is known to be associated with accuracy in diagnosing advanced fibrosis of 0.79 and 0.80, respectively, and is useful to assess for progression of liver disease and the risk of complications [36,37]. We acknowledge that TE has the highest diagnostic accuracy for cirrhosis compared to different noninvasive testing [38]; other imaging techniques, such as RMN elastography, show higher accuracy, but are often out of reach [39]. In the NASH group of patients, in our study, “progressors” had a worsening in TE results during a long follow-up, 41 patients had no changes in their liver stiffness, while a single patient showed an improvement in TE.

Our work is limited by the small sample size and by the fact that only a subgroup of patients agreed to receive a second liver biopsy. The preliminary results of studies on experimental therapeutic compounds suggest that an effective combination treatments will be available for patients with NAFLD in the coming years. In this context, case studies like ours will be impossible to be carried out; these, along with the centralized review of biopsy by the same expert pathologist, are the strength of our data.

In conclusion, according to our findings, liver histology remains the reference standard for the assessment of fibrosis progression, not only in clinical studies, but also in clinical practice, although we can expect that non-invasive imaging methods will replace it. The progression of liver disease occurs in 40% of patients with NASH after a median follow-up of almost 9 years, in the absence of any treatment. The independent predictors of progression are triglycerides higher than 150 mg/dL, and advanced fibrosis at baseline histology; however, a numerically higher number of patients with severe inflammation show progression. In waiting for non-invasive diagnostic methods able to assess both fibrosis and activity changes, histology continues to provide unique diagnostic and prognostic insights.

## Figures and Tables

**Table 1:** Characteristics of patients overall, and by progression.

Variables	Overall N = 91	Progressors N = 25	No Progressors/ Improved N = 57 *
Median Age, yrs (range)	52.5 (20–79)	53.0 (20–77)	54.0 (25–79)
Male N, (%)	43 (47.2)	9 (36.0)	26 (45.6)
Female N, (%)	48 (52.7)	16 (64.0)	31 (54.4)
BMI ≥ 30 N (%)	48 (52.7)	13 (52.0)	30 (52.6)
BMI < 30 N (%)	43 (47.3)	12 (48.0)	27 (47.4)
FIB-4 ≥ 2.67 N (%)	5 (5.5)	7 (28.0) ^	5 (8.8)
FIB-4 < 2.67 N (%)	86 (94.5)	18 (72.0)	52 (91.2)
Median PLT count, 10^3^/mm^3^	210.0 (62–402)	199.0 (100–300)	218.0 (62–402)
Median ALT (IU/mL)	62.0 (11–223)	79.1 (15–223)	71.4 (11–216)
Median AST (IU/mL)	39.5 (12–173)	57.5 (17–123)	35.0 (12–173)
Fibrosis stage N (%)			
F0–1	36 (39.5)	6 (37.0) ^	30 (50.0)
F2–4	55 (60.5)	19 (63.0)	27 (50.0)
Activity grade			
0	7 (7.6)	1 (4.0) ^^	6 (10.5)
1-2-3	84 (92.3)	24 (96.0)	51 (89.4)
Steatosis grade			
<3	65 (71.4)	19 (76.0)	38 (63.7)
3	26 (28.6)	6 (24.0)	19 (33.3)
Balloning			
1–3	60 (65.9)	21 (84.0)^	36 (63.2)
0	31 (34.1)	4 (16.0)	21 (36.8)
Portal inflammation			
<3	81 (89.0)	19 (76.0) ^	54 (97.7)
3	10 (11.0)	6 (24.0)	3 (5.3)
Diabetes Yes N, (%)	45 (49.4)	19 (76.0) °	22 (38.6)
Diabetes No N (%)	46 (50.5)	6 (24.0)	35 (61.4)
Tryglicerides			
<150	58 (63.7)	10 (40.0) ^	40 (71.4)
>150	33 (36.2)	15 (60.0)	16 (28.6)
PNPLA GG	24 (26.1) 67 (73.6)	20 (80.0) 5 (20.0)	40 (70.1) 17 (29.9)
Median KPa results	10.25 (4.2–47.2)	17.1 (6.8–47.2) °	10.8 (4.2–29.4)

* 9 patients were lost to follow up; ^ *p* < 0.05; ^^ *p* = 0.08; ° *p* = 0.004.

**Table 2:** Histological features of patients with paired biospies.

N (%)	Basal Liver Biopsy N = 26 (%)	Second Liver Biopsy N = 26 (%)
Steatosis		
Mild	7 (26.9)	5 (19.3)
Moderate	12 (46.2)	14 (53.8)
Severe	7 (26.9)	7 (26.9)
Ballooning		
0	4 (15.3)	3 (11.5)
1	22 (84.6)	23 (88.4)
Activity grade		
1	19 (73.0)	16 (61.5)
2	5 (19.3)	6 (23.1)
3	2 (7.7)	4 (15.4)
Fibrosis stage		
F0	2 (7.6)	2 (7.6)
F1	8 (30.7)	5 (19.2)
F2	9 (34.6)	9 (34.6)
F3	7 (26.9)	6 (23.1)
F4	0	4 (15.3)
Portal inflammation		
0–1	23 (89.5)	20 (76.9)
2	3 (11.5)	4 (19.2)
3	0	1 (3.8)

## Data Availability

Data supporting reported results can be found at https://zenodo.org/record/578042#.Ycy6BWnSJPw (accessed on 6 October 2022).
